# Surgical and Endoscopic Intervention for Chronic Pancreatitis in Children: The Kings College Hospital Experience

**DOI:** 10.3390/children11010074

**Published:** 2024-01-09

**Authors:** Renos M. Jeropoulos, Deepak Joshi, Bashar Aldeiri, Mark Davenport

**Affiliations:** 1 Department of Paediatric Surgery, Chelsea and Westminster Hospital, London SW10 9NH, UK; renos.jeropoulos@nhs.net (R.M.J.); bashar.aldeiri1@nhs.net (B.A.); 2 Institute of Liver Studies, King’s College Hospital, London SE5 9RS, UK; d.joshi@nhs.net; 3 Department of Paediatric Surgery, Kings College Hospital, London SE5 9RS, UK

**Keywords:** chronic pancreatitis, ERCP, endoscopic ultrasound (EUS), cystogastrostomy, pancreatojejunostomy, pseudocyst, Puestow procedure

## Abstract

Paediatric chronic pancreatitis (CP) is a rare and debilitating pathology that often requires invasive diagnostics and therapeutic interventions either to address a primary cause such as a pancreaticobiliary malunion or to deal with secondary complications such as chronic pain. Endoscopic retrograde cholangiopancreatography (ERCP) and endoscopic ultrasound (EUS) are two endoscopic modalities that have an established diagnostic role in paediatric CP, and their therapeutic utilisation is increasing in popularity. Surgical decompression of the obstructed and dilated pancreatic duct plays a role in alleviating pancreatic duct hypertension, a common association in CP. Surgery equally has a role in certain anatomical abnormalities of the pancreaticobiliary draining system, or occasionally in some CP complications such as drainage of a symptomatic pancreatic pseudocyst.

## 1. Introduction

Chronic pancreatitis (CP) is a recurrent and progressive inflammatory process that leads, if untreated, to pancreatic atrophy and loss of exocrine and endocrine pancreatic function. It presents clinically with recurrent bouts of abdominal pain, and it poses additional risks to the children that may manifest with failure to thrive, time off education, and an added hazard of opioid dependence [1,2]. There is clearly an overlap with recurrent acute pancreatitis (RAP), and many individuals with CP start this way. The aim of this study was to frame the subject using a current literature review supported by a more personal view of an institutional experience in order to summarise the common endoscopic and surgical interventions frequently advocated for CP in children and their reported outcomes.

## 2. Methods

### 2.1. Search Strategy

A preliminary search was conducted in the electronic database Pubmed^TM^ on various combinations of the terms “chronic pancreatitis”, “recurrent acute pancreatitis”, “traumatic pancreatitis”, “hereditary pancreatitis”, “pancreas divisum”, “endoscopy”, “endoscopic ultrasound”, “Puestow operation”, and “surgery” in order to evaluate the size of the literature and the suitable orientation of the terms for the main search. The main search was conducted on Pubmed^TM^ filtering records from the years 2000 to 2023 using the following terms: ((“chronic pancreatitis” OR “recurrent acute pancreatitis” OR “hereditary pancreatitis” OR pancreas divisum) AND (“endoscopy” OR “endoscopic ultrasound” OR “surgery” OR “Puestow operation”)). Titles and abstracts were screened before a full-text review of the articles.

### 2.2. Eligibility of Relevant Articles

Articles were deemed eligible for review if presenting results from surgical or endoscopic procedures performed in at least 3 children (less than 18 years of age) with acute recurrent pancreatitis, chronic pancreatitis, or hereditary pancreatitis. Exclusion criteria were as follows: (i) review articles; (ii) case series of < 3 patients; (iii) article not written in English; (iv) no intervention that was endoscopic or surgical; (v) inclusion of adult patients in data analysis.

### 2.3. Outcomes

**Figure 1:**
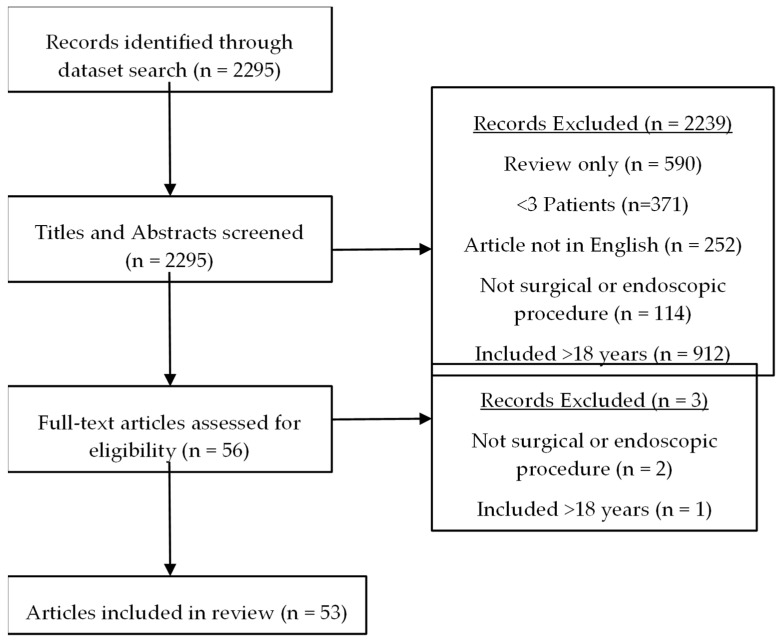
PRISMA flow chart summarising literature review.

The initial search from PubMed™ returned 2295 records. Titles and abstracts were screened to exclude 2239 records before full-article review. Three further records were excluded, leaving 53 for inclusion in this review. The screening process, including reasons for exclusion, is summarised in Figure 1.

## 3. Demographics and Aetiology

CP is not a common disease in children, but the actual estimates of its incidence are rare. For example, in one registry study from the Netherlands, the incidence of CP was ~0.5 per 100,000 persons per year in those < 20 years [3].

**Figure 2:**
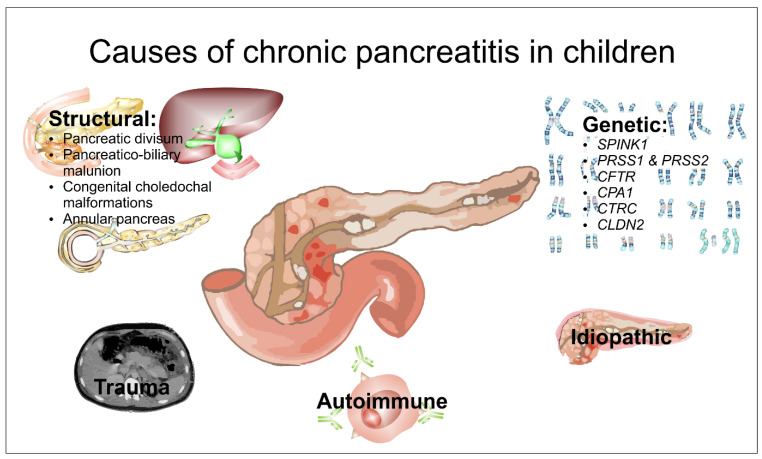
Schematic illustrating causes of chronic pancreatitis in children.

There can be many and varied aetiologies for CP, as illustrated by Figure 2. Genetic mutations (Table 1) seem to account for the majority of such cases [1,4,5] where an actual aetiology can be defined. This dates from 1996 and a report by Whitcomb et al. [6] that identified mutations in *PRSS1 (Protein Serine type 1)* in families from Kentucky and West Virginia who had children and young adults with CP, which was later confirmed in other studies [1,7,8]. Since then, other genes have been identified, including *SPINK1* (serine protease inhibitor kazal type 1) [1,9,10,11], *CFTR* (cystic fibrosis transmembrane conductance regulator) [12,13,14], *CPA1* (carboxypeptidase A1) [15], *CTRC* (chymotrypsin C) [5,16,17,18], *CLND2* (claudin-2) [19], and perhaps CELA3B (chymotrypsin-like elastase family member 3B) [20]. These children are probably best labelled as having **hereditary pancreatitis (HP)**, though many may have a de novo presentation. The spectrum of clinical features varies considerably, but there appears to be no gender disparity [8,21]. A summary of the actual mechanisms of such genetic mutations is given in Table 1. The best characterised are those of *PRSS1* and *PRSS2*, which encode for trypsinogen. These lie within a family of serine proteases, which are produced by acinar cells in an inactive form, and when secreted into the duodenum, change into their active form, trypsin. Typically, substitution of arginine for histidine at residue 117 causes the production of an unstable cationic trypsinogen that initiates autolysis and autoactivation within the pancreatic parenchyma.

Congenital anatomical anomalies of the pancreas and its draining ducts, such as **pancreatic divisum** and **pancreaticobiliary malunion**, are also implicated in the pathogenesis of CP in some children. How many is debatable, but, at least in the aforementioned INSPPIRE study, this group accounted for up to 30% of all their cases [1]. Pancreas divisum may be characterised by the failure of dorsal and ventral ducts to merge in fetal life, leading to the majority dorsal pancreas draining via the accessory duct of Santorini, while the minority ventral pancreas and uncinate retains its drainage via the main pancreatic duct and ampulla. Variations abound, and its incidence in the normal population is typically quoted at approximately 5% [21]. Various anomalies of the junction of the pancreatic and bile duct have been described, with the key feature being the creation of a “long” (variously defined) common channel between the junction and ampulla that functionally allows for the free intermixing of bile and pancreatic juices. It is a key feature of many types of choledochal malformation, including Types 1f and 1c, and Types 4f and 4c [22]. Acute pancreatitis may be a presenting feature for choledochal malformation, which may, indeed, be recurrent. However, these seldom progress to actual CP.

A degree of overlap of these kinds of anatomical anomalies and mutations, such as *SPINK1* and *PRSS1*, has been observed and attributed to the widespread genetic profiling of children with CP. For instance, 10–30% of children with malunion (*n* = 90) and pancreas divisum (*n* = 52), in one recent large Chinese study, were shown to have both *SPINK1* and *PRSS1* mutations [23].

**Trauma** (blunt or penetrating) leading to disruptions of the pancreatic duct (typically at neck level) and subsequent strictures may also play a role in a few [24,25]. Following recovery from the acute event, there may be episodes of pancreatitis due to distal duct obstruction, sometimes with stone formation and, frequently, gland atrophy. A series of 36 children with pancreatic trauma indicated a rate of up to 36% developing CP [26].

**Autoimmune pancreatitis** is still fairly ill-defined in children, accounting for ~10% of the INSPPIRE cohort [1]. They are usually characterised serologically as having raised IgG4 levels (e.g., >135 mg/dL), and histologically as having a lymphoplasmacytic infiltrate with fibrosis and typically a good response to steroids. It can be divided into Type 1 and Type 2, at least in adults, with the former having extra-pancreatic autoimmune manifestations such as sclerosing cholangitis, while the latter is only really related to inflammatory bowel disease (specifically ulcerative colitis). These patients tend to present with chronic abdominal and back pain, usually without any jaundice. Their amylase levels may be raised, and a CT scan is essentially featureless, perhaps showing pancreatic enlargement without much in the way of calcification. Our own series (*n* = 6, 2006–2013) [25] was overwhelmingly female, presented to us at a median age of 11 years with recurrent abdominal pain and, in three cases, jaundice. Two already had ulcerative colitis and all had an excellent response to steroids, though two did require ERCP and long-term stenting.

Regardless of the aetiology that triggered CP, an accumulating body of recent evidence alludes to the role of interstitial pancreatic stellate cells (PSCs) in the pathogenesis of CP [27]. The activation of these PSCs (due to oxidative stress; pro-inflammatory cytokines or many other factors) drives cytoplasmic changes, the expression of various cytoskeletal proteins (such as smooth muscle α-actin), and the transformation into a myofibroblast-like phenotype. This phenotype is capable of migration, excessive secretion of extracellular matrix (ECM), and the secretion of ECM proteins, such as matrix metalloproteinase 3 and other cytokines that are responsible for the degradation of collagen, subsequent progressive pancreatic fibrosis, and eventually pancreatic duct hypertension.

Pancreatic duct hypertension in CP is evident through duct dilatation on imaging studies such as MRCP (magnetic resonance cholangiopancreatography) or endoscopically [28]. This will, in turn, lead to further inflammation, damage to the pancreatic parenchyma, and eventually loss of exocrine and endocrine function [29], which has been shown to decline even after a single attack of acute pancreatitis [30,31]. Endoscopic and surgical procedures can be performed to facilitate the drainage of the main pancreatic duct or to address CP sequelae such as pseudocyst and stone formation.

## 4. Review of Therapeutic Interventions

### 4.1. Endoscopic Ultrasound

Endoscopic ultrasound (EUS) has gained increased popularity in recent years in the paediatric population [32,33,34,35,36], following its first report in 1998 on a series of 18 children from the Cochin Hospital, Paris [37]. EUS has been shown to be more sensitive at diagnosing anatomical variations and radiolucent stones in the pancreatobiliary system and can even evaluate CP earlier, in terms of disease progression, than MRCP [38,39].

In addition to its use in diagnosing pancreatobiliary disorders [40,41,42,43], EUS now has a major therapeutic role in guiding endoscopic transmural drainage procedures such as cyst gastrostomies and cyst enterostomies [44,45] in children with pancreatic pseudocysts or para-pancreatic fluid collections [46,47,48]. It has the advantage of measuring the distance between the gastric/duodenal wall and the para-pancreatic fluid collection, while the use of Doppler US ensures there are no interposed blood vessels in between. There are a few single-centre series reported, with encouraging results and relatively few complications [32,33].

EUS may also be used safely to guide coeliac plexus regional block in children with CP and intractable abdominal pain. For instance, Keane et al. reported improvement in pain symptoms in a 17-year-old girl with intractable pain who received a coeliac plexus block under general anaesthesia, though not sufficiently to stop her existing medications [33]. Other indications include EUS-guided biliary puncture and rendezvous procedures which remove the need for external puncture [49].

The combination of EUS and ERCP during a single setting has also been shown to be beneficial. Case series from Belgium [32] and London [33] have reported success in utilising the added sensitivity of ultrasound to identify stones prior to attempting ERCP, sphincterotomy, and stone extraction in paediatric CP.

### 4.2. Endoscopic Retrograde Cholangiopancreatography (ERCP)

Pancreatitis is a common indication for ERCP in children [28,32,33,50,51,52,53,54,55]. In our opinion, ERCP is an essential diagnostic tool with which to obtain accurate pancreatograms and cholangiograms in order to assess likely aetiology and describe anatomy [56]; but also, in many cases, it has an important therapeutic role. This is particularly true for the identification of pancreaticobiliary malunion and pancreas divisum and the evaluation of pancreatic duct strictures (Table 2).

Tertiary centres with high volumes of ERCP have published promising data on the use of adult scopes for children as young as one year of age, with a success rate of cannulation of approximately 90% [50,51,53,57]. The risk of failed cannulation of the major or minor pancreatic papilla increases with decreased body weight [28,52].

The therapeutic scope of ERCP in paediatric CP is ample. In cases of CP with intractable pain, ERCP can be considered for the evaluation of a dilated pancreatic duct and decompression with a stent and sphincterotomy of either the major or minor papilla [58,59]. Pain control and reduced analgesic intake are the main outcome measures of successful decompression, and are considered prerequisites in some centres prior to offering a more invasive surgical decompression procedure [60]. Kohoutova et al. reported a series of 38 CP children who underwent an ERCP, sphincterotomy, and stent placement for intractable abdominal pain, with a median follow-up of 7 years, with 66% of children reported as pain-free and with a significant reduction of analgesia use in all children post-ERCP [28]. However, where decompression was achieved via stent insertion, stents would need to be changed periodically [54,61]. Nearly three quarters of the patients in the aforementioned series underwent repeated ERCP [28], indicating the temporary nature of this intervention. This limits the insertion of stents to patients who are amenable to close follow-up and exposes children to repeated interventions under sedation or general anaesthesia [33].

In patients with pancreatic duct stenosis, the placement of fully covered metallic stents (FcSEMS), as opposed to plastic stents, seems to reduce the need for further ERCP and stent changes [62,63]. In addition, FcSEMS can remodel strictures and provide more durable patency. Where stents are used to decompress pancreatic duct hypertension in CP, we believe that ERCP and stenting are a temporary measure with which to alleviate symptoms and, more importantly, assess the likelihood of success of a surgical pancreatojejunostomy.

**Table 2:** Summary of ERCP-diagnosed chronic pancreatitis (*n* = 59): Kings College Hospital experience (2008–2018) [63].

Aetiology of Chronic Pancreatitis (*n* = 59)		Diagnostic Findings
Idiopathic	*n* = 11 (19%)	Duct dilatation, *n* = 52 (88%) Gland atrophy, *n* = 16 (27%) Fluid collection, *n* = 7 (12%)
Gallstones	*n* = 22 (37%)	
Hereditary	*n* = 10 (17%)	
Pancreatobiliary junction anomalies	*n* = 8 (14%)	
Autoimmune	*n* = 8 (14%)	
**No. of ERCPs (*n* = 126)**		
Stent placement	*n* = 78 (62%)	**Indication**:
	Plastic, *n* = 72 (92%) Metal, *n* = 6 (8%)	Duct stricture, *n* = 57 (73%) Leak or fluid collection, *n* = 21 (27%).

ERCP combined with pancreatoscopy plus electrohydrolytic lithotripsy (EHL) seems to be superior in patients with pancreatic duct stones. Cholangioscopy with EHL has been performed in our centre since 2018. The major limitation is the diameter of the bile duct or pancreatic duct as the cholangioscope/pancreatoscope has an outer diameter of 3.3mm. Similarly, the simultaneous use of EUS and ERCP has been shown to improve stone extraction success [32,33].

ERCP is still considered to carry a significant post-procedure complications risk, with the most common being post-ERCP pancreatitis at 2–9% [51,52,53]. Other complications include infections such as cholangitis and cholecystitis, post-ERCP bleeding, and the risks carried by anaesthesia [28,33]. With repeated ERCP procedures, children have a higher cumulative risk of the above. However, in experienced centres with the appropriate multi-disciplinary team support, ERCP remains integral to the management of CP.

The largest experience to date of ERCP in children appears to come from Hyderabad in India [64]. Here, 221 ERCPs (therapeutic, *n* = 157) were performed on 172 children, mostly for CP (83%). Sphincterotomy, stenting, and EHL were used liberally, with a reported improvement in symptoms in about 83%. Keane et al., in a systematic review, noted that the lowest complication rates were seen in CP when the ERCP was done by “adult” endoscopists and those in high-volume centres [33]. In their own series, no complications were attributed to ERCP.

### 4.3. Surgical Intervention in Children with Chronic Pancreatitis

#### 4.3.1. Pancreatojejunostomy

Modern surgical management of CP and its complications dates back to the mid-20th century. The ultimate aim of surgery in CP is to preserve the pancreatic parenchyma and its endocrine and exocrine function. This is achieved by ensuring adequate pancreatic duct drainage to counteract the devastating effect of duct hypertension, subsequent enzymatic extravasation, and autolysis of pancreatic parenchyma. There is little evidence to support the superiority of any of the drainage procedures; however, the wider the anastomosis between the jejunal loop and the pancreatic duct, the better the drainage that will be achieved and the less likely will be for a future stricture will develop. Merlin Du Val first introduced the concept of retrograde pancreatic duct drainage via a caudal pancreatojejunostomy in 1954 [65]. This, however, suffered from anastomotic stricture and poor drainage. Nowadays, an extension of the concept of retrograde pancreatic duct drainage, usually referred to as the longitudinal pancreatojejunostomy (LPJ) or the modified Puestow procedure, is the most performed procedure in paediatric CP [2]. Charles Puestow (1902–1973) originally described a splenectomy, distal pancreatectomy, and invagination of the opened duct in the body and tail of the pancreas into a Roux loop and used this principally for alcohol-induced CP in adults [66]. A couple of years later, Partington and Rochelle introduced the concept of suturing the Roux loop longitudinally into the opened pancreatic duct in the head and tail of the pancreas, often without the need to perform a splenectomy [67]. The most recent modification of the technique is known as the Frey procedure [68], though there are few reports of this in children [69,70]. This requires a Kocher mobilisation of the head of the pancreas to permit palpation and assessment of the fibrotic infiltration and stone formation within the head and may involve a local resection of pancreatic parenchyma. All of the splayed ductal system is then drained in a retrograde fashion via a Roux loop.

There is a relatively small body of literature for surgery in paediatric CP. Most evidence is derived from small series from individual centres, including our own published in 2016 [60], or a cumulative retrospective review of multicentre studies. A recent systematic “scoping” review identified about 200 paediatric LPJ procedures reported since the year 2000, with the largest of these being a series from Kolkata, India, of 26 cases of LPJ together with 25 of Frey’s modification of LPJ [70,71]. Ford et al. [60] reported our original experience with a series of eight children with established CP who were offered definite surgical drainage (LPJ) following a therapeutic trial of ERCP and stenting. There was a marked reduction of pain post-op, as well as the normalisation of weight and subsequent growth.

The commonest indications for LPJ have included persistence of symptoms despite previous sphincteroplasty or ERCP, failed ERCP and stenting, pancreatic strictures, and patients opting for upfront surgical management of CP [24,54,61,72,73,74,75].

Our strategy of modern management of CP is based on adequate symptoms and pain control in the acute phase, maintenance of normal nutrition, and correction of any endocrine or exocrine deficiencies. When any of these elements are not controlled, operative intervention should be considered with the primary aim of offering lasting pain relief. We adopted a multimodal approach wherein we used ERCP and stenting to provide a therapeutic trial to assess the possible benefit of a definitive duct drainage procedure [60].

**Figure 3:**
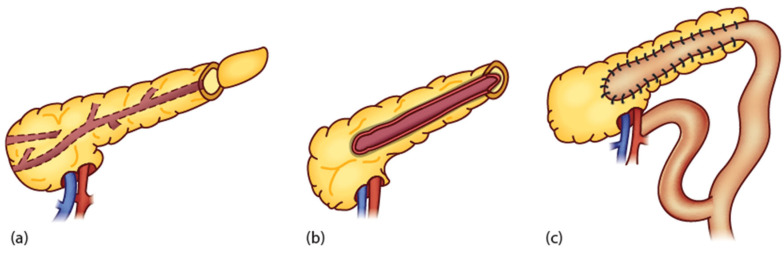
Schematic illustration of longitudinal pancreatojejunostomy (Puestow procedure) (reproduced with permission from Davenport M., “Chronic Pancreatis In Surgery of the Liver, Bile Ducts and Pancreas in Children” (eds). Davenport M, Heaton ND, Superina R, CRC Press 2016) [25]. (**a**) Mobilisation of tail of pancreas from splenic hilum with freeing of splenic vein and artery. Amputation of the tail to identify the dilated pancreatic duct. Sometime, an on-table ultrasound may help to identify the dilated duct more proximally. (**b**) Laying open the duct along the body to the neck. (**c**) Creation of an isograde Roux loop and pancreatojejunostomy.

**Figure 4:**
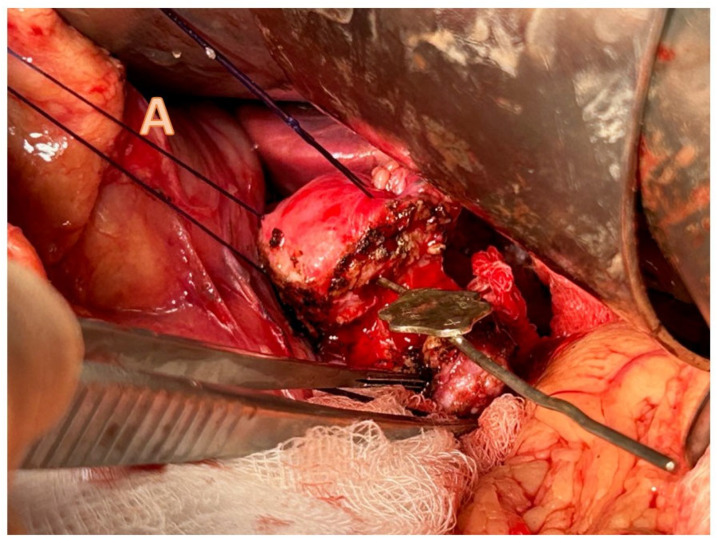

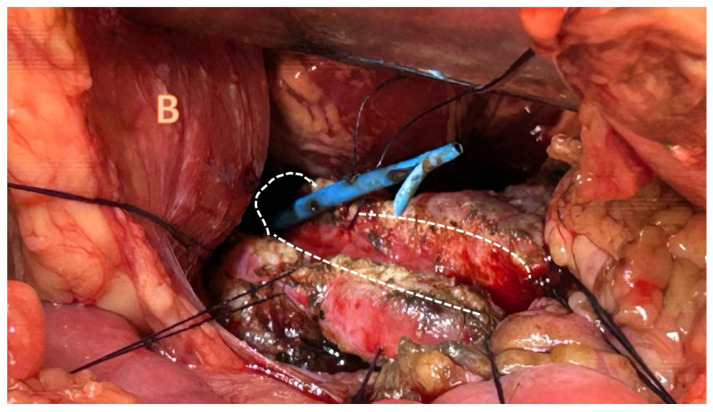
Longitudinal pancreatojejunostomy (Puestow procedure) [25]. Operative photographs showing (**A**) initial amputation of tail and identification of distal duct using a lacrimal probe; (**B**) opening of duct along length exposing indwelling stent. The dotted line shows margins that will form the longitudinal anastomosis.

Figure 3 and Figure 4 illustrate the schematic and operative approach of a longitudinal pancreatojejunostomy [25].

The short-term outcomes of PJ are generally favourable. Most series report a reduction in frequency, or sometimes complete resolution, of bouts of pancreatitis; reduction in pain and chronic analgesia use; and normalisation of weight post-procedure [24,60,72,75,76]. However, the long-term consequences of CP, such as a requirement for pancreatic enzyme supplementation or development of insulin-dependent diabetes, do not seem to reduce with an LPJ [24,60]. Data obtained from adult patients with hereditary pancreatitis suggest that pancreatic insufficiency develops after a LJP in the long-term, even with evidence of a patent anastomosis [77,78]. Similarly, pain resolution in adults after a PJ in the long term does not seem to be sustainable [79]. Whether this is due to an ongoing inflammatory process in the perineural tissue in the pancreas region or to a relapse of the original pathology seems speculative.

The main complications of an LPJ is an early anastomotic leak or the future development of an anastomotic stricture. A recent systematic review identified five such cases of anastomotic strictures after an LPJ in over 150 paediatric PJ procedures [2]. The incidence is likely much higher than this, but long-term follow-up sufficient to reliably inform outcomes has not been achieved. Further reported complications included prolonged paralytic ileus, respiratory complications, and acute pancreatitis [2,60,61,80].

A pancreatojejunostomy that incorporates the common bile duct as an hepaticojejunostomy has also been reported to address pancreaticobiliary malunion with dual pathology of pancreatitis and jaundice [28,50]. Nevertheless, due to its rarity, the long-term outcome of this approach is unknown. We have performed this in two children with obstructive jaundice and CP using two separate Roux loops with excellent results.

Though the description here uses an open approach, and this is the most common method, at least in children, it should be noted that laparoscopic LPJ has been reported. So, Zhang et al. [81] from Beijing reported four cases, with the youngest being six years of age. They modified and simplified the technique by doing the initial jejunojejunostomy extracorporeally and only did a single-layer pancreatic anastomosis. There are others [82,83], and to overcome the clear technical anastomotic difficulties, some centres have reported use of a robot [84,85], though at present only in adults. Some centres compromise the anastomotic technique considerably to facilitate a minimally invasive technique (e.g., using a Witzel modification) [86].

#### 4.3.2. Other Surgical Options

The analgesic effect of pancreatic duct decompression in CP has been seen to diminish over time in adults, and it is thought to be due to the persistence of neuropathic pain [87]. A resection of the most diseased pancreatic parenchyma has been shown to provide pain relief, at least in short term, in children with CP. Chromik et al. [88] reported a series of six children with CP that have had partial pancreatectomies. Where this led to a transection or a discontinuation of the pancreatic duct, a lateral or a longitudinal PJ was fashioned to drain the tail of the pancreas. Four of the six children so treated did not require analgesia at follow-up, and one reported a markedly reduced requirement. In this series, none of the patients exhibited new pancreatic insufficiency; however, the two patients that were already established on enzyme supplementation continued post-op. Smaller case series reported partial pancreatic resections to treat strictures that prevented ERCP cannulation, or in the presence of multiple pancreatic duct calculi [24,54,80,89].

#### 4.3.3. Total Pancreatectomy and Islet Cell Transplant

Where pain relief has not been achieved despite adequate drainage or partial resection procedures, a total pancreatectomy can be considered. Case series from Minnesota have reported success in reducing the opioid burden in children with CP by performing a total pancreatectomy with islet cell autotransplantation (TP/IAT) [90,91,92]. Most of the patients receiving TP/IAT had previously undergone ERCP or another surgical procedure without resolution of symptoms. Of the 15 patients followed-up in one series following TP/IAT, 10 reported no pain at all, while 4 reported pain improvement [90]. However, almost half of those patients were insulin-dependent at one year post-IAT, and 14 out of 15 patients were reported to be on pancreatic enzymes on a regular basis. In another series of 14 children with CP who underwent TP/IAT, only 29% progressed to insulin independence [92]. There is some evidence to suggest that the earlier TP/IAT is performed, the lower the rates of long-term endocrine insufficiency [93], which is attributed to the higher yield of islet cells harvested. Despite the likelihood of insulin dependence, quality of life questionnaires show an improvement of patient-perceived mental and physical health following TP/IAT [92], which puts into perspective the negative impact of chronic pain from CP.

## 5. Discussion

CP in children has a variety of causes and requires a holistic approach to treatment. The mainstay of medical management is ensuring adequate analgesia, commonly with opioids, and maintaining nutrition either enterally or parenterally. Failure to thrive is not uncommon for children with CP, so ensuring adequate caloric intake is paramount. Pancreatic insufficiencies, as a consequence of CP, can be treated with enzyme supplementation or insulin as necessary. Decompression of the pancreatic duct can be achieved endoscopically or surgically, with pancreatic resections being an option, as discussed above.

A multi-disciplinary team (MDT) must be used to treat children with CP, given the complexity of the medical needs of such patients, and include gastroenterologists, radiologists, endocrinologists, surgeons, pain specialists, psychologists, and dieticians [94]. It is also most likely that sufficient experience will only be available in tertiary centres in the case of this relatively rare condition.

What is not known is the actual long-term (>10 years) outcome of surgical and endoscopic drainage procedures in patients with CP with an onset during childhood. We believe that early intervention may have the benefit of preventing sequelae of on-going disease in addition to alleviating symptoms such as chronic pain.

Most surgical interventions that are described in the literature are sought after previous interventions have failed to provide disease control. ERCP alone predisposes a child with CP to multiple hospital admissions and anaesthetic episodes for stent changes every few months. By offering definite surgical management of pancreatic drainage early, pain, as well as repeated sedation/anaesthesia, can potentially be avoided. Surgical techniques may improve in the future with a greater use of laparoscopic techniques (and perhaps robot-assistance), but their functionality and results can only remain the same.

Long-term follow-up is essential. A diagnosis of Type 3c diabetes should be looked for (i.e., pancreatic islet dysfunction and islet loss as a result of diseases of the exocrine pancreas) and can be made if the following criteria are identified: the presence of pancreatic pathology on imaging together with exocrine pancreatic insufficiency (with the absence of autoimmune markers suggestive of type 1 diabetes). A decrease in β-cell function, absence of insulin resistance, loss of incretin secretion, and low fat-soluble vitamin concentrations can further support the diagnosis [95]. Screening with measurement of glycated hemoglobin A1c (HbA1c) or fasting glucose are reasonable [96].

Management of Type 3c diabetes involves optimising pancreatic enzyme replacement therapy. They are at an increased risk for hypoglycaemia due to a lack of counter regulation. Although insulin is often the treatment of choice, mild hyperglycaemia (HbA1c < 8%) can be treated with metformin, which has also been recommended [96].

CP also confers a risk of the development of malignancy in the damaged pancreatic remnant, although by how much is far from clear. One Chinese study estimated a relative risk of about 20 compared to national controls, with a much greater risk if they were smokers [97]. However, they suggested that younger age at onset was in some way protective. A further study from Chengdu, China, in post-operative adult patients with CP showed that a further risk factor was the de novo development of endocrine deficiency [98]. Kirkegård et al. [99] performed a meta-analysis of 13 eligible studies to try to quantify the risk and showed a 16-fold increased risk of the development of cancer within 2 years of the diagnosis of CP, declining to 4-fold if the diagnosis of CP was made > 9 years previously. Those with diagnosed hereditary pancreatitis are, however, at special risk of this complication, and of course make up a significant proportion of children with CP. Their risk has been estimated at up to 70 times that of a normal population, and they should certainly enter a long-term screening programme during adulthood with alternating MRCP and EUS surveillance [4,95,98,99,100].

## 6. Conclusions

CP in children is a rare but debilitating condition, and long-term outcomes of interventions are not yet known. Early intervention for pancreatic duct drainage should be considered in tertiary centres with an MDT approach with an aim to minimise morbidity and the progression of disease.

## Figures and Tables

**Table 1:** Reported genetic mutations in chronic pancreatitis in children.

Gene	Mechanism	Geography	References
***PRSS1* and *PRSS2*** Encoding Protein Serine type 1 and type 2	Production of an unstable cationic trypsinogen that exhibits reduced activity, autolysis, and autoactivation	N. America Germany Australia	Whitcomb et al. (1996) [6] Gorry et al. (1997) [7] Kumar et al. (2016) [1] Wu et al. (2022) [8]
***SPINK1* (*p.N34S*)** Encoding Serine Protease Inhibitor Kazal type 1	Mutations reduce the inhibition of unregulated and prematurely activated trypsin, causing cellular damage within the parenchyma	China, Korea—IVS 3 + 2 T > C Europe—p.N34S N. America—p.N34S	Witt et al. 2000 [9] Chen et al. 2000 [10] Kumar et al. (2016) [1] Liu et al. (2017) [11]
***CFTR*** Encoding Cystic Fibrosis Transmembrane Conductance Regulator	↓ ductal fluid and ↓ bicarbonate secretion, leading to ↓ intraluminal pH, ↓ washout of the digestive enzymes, and more viscous protein-rich ductal fluid	Europe N. America China/Taiwan	Sharer et al. (1998) [12] Cohn et al. (1998) [13] Chang et al. (2007) [14]
***CPA1*** Encoding Carboxypeptidase A1	Misfolding CPA1 phenotype protein resulting in pancreatic endoplasmic reticulum (ER) stress	Germany	Witt et al. (2013) [15]
***CTRC*** Encoding Chymotrypsin C	Impaired trypsin degradation	Europe China Asia	Rosendahl et al. (2008) [16] Masson et al. (2008) [17] Wang et al. (2013) [18] Koziel 2015 [5]
***CLDN2*** Encoding Claudin 2	Atypical localization of CLDN2 protein leading to alterations in pancreatic ductal fluid composition and/or imbalance in calcium homeostasis	Europe N. America	Whitcomb et al. (2015) [19]
***CELA3B*** Encoding chymotrypsin like elastase 3B	Uncontrolled proteolysis of trypsin due to upregulation of CELA3B	N. America	Moore et al. (2019) [20]

## Data Availability

Not applicable.

## Abbreviations

*CELA3B*	Chymotrypsin-like elastase family member 3B
*CFTR*	Cystic fibrosis transmembrane conductance regulator
*CPA-1*	Carboxypeptidase A1
*CLDN2*	Claudin-2
*CTRC*	Chymotrypsin C
ECM	Extracellular matrix
ERCP	Endoscopic retrograde cholangiopancreatography
EUS	Endoscopic ultrasound
INSPPIRE	International Study Group of Pediatric Pancreatitis: In Search for a CuRE
MDT	Multi-disciplinary team
MRCP	Magnetic resonance cholangiopancreatography
*PRSS1* and *PRSS2*	Protein serine type 1 and type 2
*SPINK1*	Serine protease inhibitor Kazal type 1
